# Factors Associated With Intention of Future Pregnancy Among Women Affected by the Fukushima Nuclear Accident: Analysis of Fukushima Health Management Survey Data From 2012 to 2014

**DOI:** 10.2188/jea.JE20180015

**Published:** 2019-08-05

**Authors:** Aya Goto, Yusuke Tsugawa, Keiya Fujimori

**Affiliations:** 1 Center for Integrated Science and Humanities, Fukushima Medical University, Fukushima, Japan; 2 Division of General Internal Medicine & Health Services Research, David Geffen School of Medicine at the University of California, Los Angeles, CA, USA; 3 Department of Obstetrics and Gynecology, Fukushima Medical University School of Medicine, Fukushima, Japan

**Keywords:** mothers, fertility, family planning policy, radiation, Fukushima nuclear accident

## Abstract

**Background:**

Little is known about the association between the anxiety toward the effects of radiation on reproduction caused by the Fukushima nuclear accident and the birth rate of people in Fukushima. Therefore, we examined changes and associated factors of future pregnancy intention among mothers in Fukushima Prefecture.

**Methods:**

Using data from three postal surveys among women who registered their pregnancies in the prefecture (*N* = 6,751 in 2012, *N* = 6,871 in 2013, and *N* = 6,725 in 2014), we analyzed the factors associated with women’s intention of future pregnancy using multivariable logistic regression models.

**Results:**

The proportion of mothers with pregnancy intention increased from 53.5% in 2012 to 57.9% in 2014, especially among multiparas (*P* for trend <0.001). Factors inversely associated with pregnancy intention of both groups were older maternal age (adjusted odds ratio [aOR] 0.92 for primipara and 0.87 for multipara), poor subjective health (aOR 0.75 and 0.81, respectively), and presence of depressive symptoms (aOR 0.71 and 0.79, respectively) (*P* < 0.01 for all items). In addition, not living with husband (aOR 0.24), dissatisfaction with obstetrical care (aOR 0.89) and child abnormalities (aOR 0.72) were inversely associated with pregnancy intention among primiparas, while receiving infertility treatment (aOR 2.05) was positively associated among multiparas (*P* < 0.01 for all items). A separate analysis of 2012 and 2013 data showed that concern about radiation contamination of breast milk was associated with pregnancy intention among primiparas (aOR 0.61, *P* < 0.001).

**Conclusions:**

Mothers’ concern about radiation was associated with lower pregnancy intention, especially among primiparas. Providing quality obstetrical and mental health care and parenting support may be the keys to maintaining the temporal increase in fertility.

## BACKGROUND

Japan’s low birth rate has long been a societal concern, leading to a rapidly aging population with a shrinking working-age population. In 2016, Japan’s total fertility rate was 1.44, which was far below the replacement fertility rate of 2.1.^1^ Moreover, our recent analysis of Japanese government statistics on live births, stillbirths, and induced abortions found that there was no upward trend in fertility since 1974, except for the abortion rate showing a slight increase during 1996–2002.^2^ The Fukushima Nuclear Power Plant accident occurred in 2011 after the Great East Japan Earthquake and Tsunami and caused region-wide radiation contamination. The low birth rate could, in theory, deteriorate because of anxiety over the effects of radiation on reproduction caused by the Fukushima nuclear accident, especially given that fetuses and children are more susceptible to radiation than adults. A previous study found that the number of births declined in Japan after the 2011 disaster at the national level (ie, irrespective of proximity to the affected area).^3^ Given the strong interest in increasing the birth rate in Japan and in other developed countries, and the fact that any country with nuclear power plants can potentially be affected by a nuclear accident like Japan, it is critically important to understand how a nuclear accident and radiation contamination affect women’s perceptions and decisions about future pregnancy.

Fukushima Medical University immediately launched a prefecture-wide cohort survey (the Fukushima Health Management Survey [FHMS]) in order to estimate the level of external exposure to radiation and to assess and support community health.^4^ A survey among evacuees conducted as a part of the FHMS reported that one-third believed that genetic effects caused by radiation were very likely to occur, and such concern about radiation risks was associated with psychological distress.^5^ In the face of a nuclear disaster, mothers of young children were also identified as one of the groups at greatest risk of negative emotional and mental health consequences.^6^ Using another set of FHMS data among pregnant women, we reported that one in four mothers who delivered within 1 year after the disaster screened positive for depression,^7^ and their common concerns were radiation effects on fetuses and infants.^8^

The current analysis builds on these findings by examining the changes of future pregnancy intention among mothers living in Fukushima who responded to the FHMS survey, reasons behind such intentions, and associated factors in order to better understand how women make fertility decisions under such circumstances.

## METHODS

### Setting and procedure

The Pregnancy and Birth Survey of the FHMS has been conducted every year since the disaster, targeting women who registered their pregnancies during a specified period in each year.^9^ Every pregnant woman in Japan is required to register her pregnancy in order to receive free access to antenatal care and well-child visits.

### Participants and recruitment

The annual survey targeted women who registered their pregnancies during 1 year from August 1 of the previous year to July 31 of the current year. Lists of registered women were obtained from all municipalities in the prefecture. Reminders were sent once for the 2012 survey (June 28, 2013), twice for the 2013 survey (May 10 and July 31, 2014), and once for the 2014 survey (July 24, 2015). Data collection continued for about 1 year (until November 30, 2013 for the 2012 survey, December 26, 2014 for the 2013 survey and December 18, 2015 for the 2014 survey).

**Figure 1.**
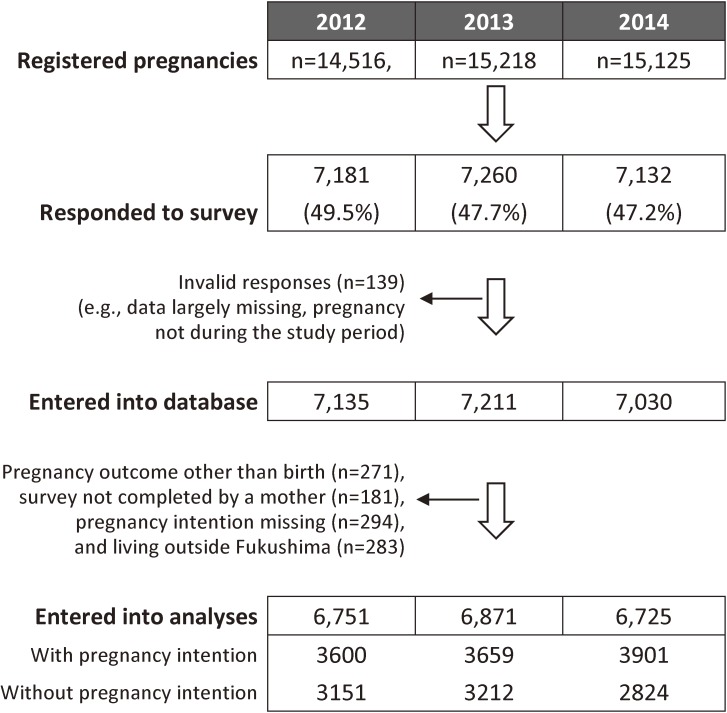
Flow diagram of participants

Of the 14,516, 15,218 and 15,125 identified women, 7,181 (49.5%), 7,260 (47.7%), and 7,132 (47.2%) returned the questionnaire in 2012, 2013 and 2014, respectively (Figure 1). The present analysis focused on mothers who lived in Fukushima, filled out the questionnaire by themselves, had live births, and completed the questionnaire item on pregnancy intention (6,751 in 2012, 6,871 in 2013, and 6,725 in 2014). The number of duplicate (with other years) live births in each year was 845 (13% of 6,751) in 2012, 308 (4% of 6,871) in 2013, and 871 (13% of 6,725) in 2014.

### Data management and sources

The FHMS database is computerized and managed centrally by the Radiation Medical Science Center of the FHMS. In the current study, we analyzed data from 2012, 2013, and 2014 since the question regarding future pregnancy intention was introduced in 2012.

### Data items

The analysis focused on pregnancy intention and its associated factors. Pregnancy intention was assessed using a single question: “Are you planning to become pregnant in the future?”. “Yes” was classified as with and “no” as without intention of future pregnancy. A similar question asking women about future pregnancy intention with a binominal answer option is used in the National Fertility Survey in Japan and the National Survey of Family Growth in the United States.^10^^,^^11^ Answer options for a further question about reasons for not planning pregnancy were also extracted from the National Fertility Survey. Answer options for an adjunct question about service demands, asked to mothers with pregnancy intention, were decided among FHMS members by referring to government surveys on parenting.^12^

Potential associated factors for pregnancy intention included in the analyses were: maternal factors (age at pregnancy, medical history, residential region, subjective health, depression symptoms); living arrangement (current evacuation, living with husband), obstetrical and parenting factors (pregnancy history, fertility treatment, satisfaction with obstetrical care, and maternal confidence); and infant characteristics (sex and abnormalities at birth).

Residential regions were classified into three categories: the most populated central region with relatively higher radiation levels, the coastal region closest to the nuclear power plant and partially designated as an evacuation zone, and the mountainous region farthest from the nuclear power plant. To assess depressive symptoms, a standard two-item screening measure, asking about depressed mood and anhedonia over the past month, was administered.^7^ Mothers who answered yes to one or both of these questions were classified as positive for depressive symptoms. Maternal confidence was assessed using a single question: “Are there any moments when you don’t feel confident about child rearing?”.^13^ The response codes were “yes,” “unsure,” and “no,” with “yes” or “unsure” classified as lack of confidence in child rearing.

In addition to current evacuation at the time of the survey (designated and self-determined), we included “concern about radiation contamination of breast milk” as a Fukushima-specific factor. This was defined as bottle feeding their babies because of radiation concerns (vs breast feeding and bottle feeding as a result of lack of breast milk or other reasons), as in our previous analysis on maternal mental health.^13^ Of note, the question about infant feeding was asked only in 2012 and 2013, not in 2014.

### Statistical analysis

The Wilcoxon-type test for trend developed by Cuzick was used to compare proportions across the 3-year study period.^14^ With regard to the analysis of parity-specific factors associated with pregnancy intention, we used a *t* test for continuous variables and a chi-squared test for categorical variables. Variables significantly associated with pregnancy intention were entered into a multivariable analysis using logistic regression stratified by parity. Another set of multivariable analyses were conducted by limiting data to the first 2 years and by focusing on the item on concern about radiation adjusting for factors analyzed in the above-mentioned analysis of parity-specific factors.

A conservative *P*-value of less than 0.01 was considered statistically significant given the large sample sizes and the number of comparisons. All statistical analyses were conducted using STATA, version 10 (Stata Corp, College Station, TX, USA).

### Ethical considerations

The ethics committee of Fukushima Medical University approved this study (No. 1317). The survey aims were explained to all respondents in a cover letter that was sent out with the questionnaire. By responding to the survey, participants were considered to have consented to participation.

## RESULTS

**Figure 2.**
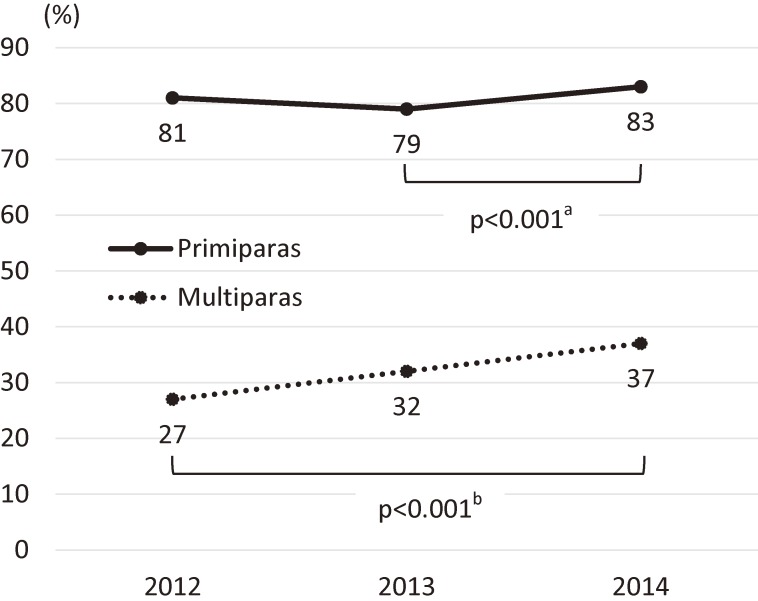
Respondents’ pregnancy intention by parity. ^a^Two-sample test of proportion was used to compare data for 2013 and 2014. ^b^Nonparametric test for trend was used to compare data across the 3 years.

The mean maternal age was 30.6 years, and 46.5% were primiparas. The overall proportion of those with pregnancy intention was 53.3% in 2012, 53.3% in 2013, and 58.1% in 2014, and was 81.1% among primiparas and 32.1% among multiparas. Parity-specific trends showed a significant increase from 2013 to 2014 among primiparas (*P* < 0.001) and across the 3 years among multiparas (*P* for trend <0.001) (Figure 2).

Comparing respondents’ characteristics by pregnancy intention (Table 1), primiparas with pregnancy intention were significantly younger, had no medical history, had good subjective health, had no depressive symptoms, lived in a mountainous region, had not been evacuated, lived with their husband, were satisfied with obstetrical care, were less confident in childrearing and were more likely to have children with abnormality at birth (*P* < 0.01). Among multiparas, those with pregnancy intention were significantly younger, had good subjective health, had no depressive symptoms, lived with their husband, had received infertility treatment, were satisfied with obstetrical care and were less confident in childrearing (*P* < 0.01). Multivariable analyses (Table 2) found that factors associated significantly with pregnancy intention among primiparas were survey year (aOR 0.83 for 2013), maternal age (aOR 0.92 for 1-year increase), subjective health (aOR 0.75 for poor), depressive symptoms (aOR 0.71 for positive symptoms), living with husband (aOR 0.24 for not living together), satisfied with obstetrical care (aOR 0.89 for not satisfied), and child abnormality at birth (aOR 0.72 for having abnormality). Significant factors for multipara were survey year (aOR 1.28 for 2013 and 1.68 for 2014), maternal age (aOR 0.87 for 1-year increase), subjective health (aOR 0.81 for poor), depressive symptoms (aOR 0.79 for symptoms positive), and infertility treatment (aOR 2.05 for receiving treatment).

**Table 1.** Respondents’ characteristics and differences by parity and pregnancy intention

Characteristics	Total^a^ (*n* = 20,347)	*N* (%) or *Mean (SD)* Pregnancy intention among primiparas		*P* value^b^	*N* (%) or *Mean (SD)* Pregnancy intention among multiparas		*P* value^b^
	
		No (*n* = 1,788)	Yes (*n* = 7,664)		No (*n* = 7,399)	Yes (*n* = 3,496)	
**Maternal characteristics**							
Age at the time of pregnancy, years	***30.6 (5.0)***	*31.0 (6.1)*	*28.9 (4.8)*	<0.001	*32.6 (4.6)*	*29.8 (4.4)*	<0.001
Medical history							
No	**14,726 (72.6)**	1,190 (67.0)	5,450 (71.3)	<0.001	5,446 (73.8)	2,640 (75.8)	0.026
Yes	**5,558 (27.4)**	586 (33.0)	2,193 (28.7)		1,935 (26.2)	844 (24.2)	
Subjective health							
Good	**19,576 (96.3)**	1,669 (93.4)	7,447 (97.2)	<0.001	7,066 (95.5)	3,394 (97.1)	<0.001
Poor	**762 (3.8)**	118 (6.6)	212 (2.8)		332 (4.5)	100 (2.9)	
Depression symptoms							
No	**15,363 (75.6)**	1,192 (66.8)	5,756 (75.3)	<0.001	5,645 (76.4)	2,770 (79.4)	<0.001
Yes	**4,947 (24.4)**	592 (33.2)	1,888 (24.7)		1,748 (23.6)	719 (20.6)	
**Residential characteristics**							
Residential region							
Central	**12,784 (62.8)**	1,139 (63.7)	4,880 (63.7)	0.009	4,612 (62.3)	2,153 (61.6)	0.445
Coastal	**4,913 (24.2)**	463 (25.9)	1,809 (23.6)		1,797 (24.3)	844 (24.1)	
Mountainous	**2,650 (13.0)**	186 (10.4)	975 (12.7)		990 (13.4)	499 (14.3)	
Current evacuation							
No	**18,813 (93.9)**	1,628 (93.2)	7,174 (94.8)	0.009	6,804 (93.6)	3,207 (93.2)	0.331
Yes	**1,213 (6.1)**	119 (6.8)	396 (5.2)		462 (6.4)	236 (6.9)	
Living with husband							
Yes	**19,058 (93.7)**	1,469 (82.2)	7,238 (94.4)	<0.001	7,000 (94.6)	3,351 (95.9)	0.005
No	**1,289 (6.3)**	319 (17.8)	426 (5.6)		399 (5.4)	145 (4.2)	
**Obstetrical and parenting characteristics**							
Infertility treatment							
No	**19,069 (94.0)**	1,611 (90.7)	7,030 (92.0)	0.068	7,115 (96.4)	3,313 (95.1)	0.001
Yes	**1,212 (6.0)**	165 (9.3)	609 (8.0)		267 (3.6)	171 (4.9)	
Satisfaction with obstetrical care							
Yes	**17,341 (85.4)**	1,425 (80.0)	6,542 (85.5)	<0.001	6,315 (85.5)	3,059 (87.7)	0.002
No	**2,971 (14.6)**	357 (20.0)	1,109 (14.5)		1,075 (14.6)	430 (12.3)	
Maternal confidence							
Yes	**9,007 (44.8)**	521 (29.6)	2,698 (35.6)	<0.001	3,957 (54.1)	1,831 (52.8)	0.234
No	**11,109 (55.2)**	1,237 (70.4)	4,873 (64.4)		3,364 (46.0)	1,635 (47.2)	
**Infant characteristics**							
Sex							
Boy	**10,414 (51.4)**	889 (50.0)	3,933 (51.5)	0.251	3,785 (51.4)	1,807 (51.8)	0.636
Girl	**9,851 (48.6)**	888 (50.0)	3,698 (48.5)		3,586 (48.7)	1,679 (48.2)	
Abnormalities at birth^c^							
No	**17,482 (87.6)**	1,461 (83.8)	6,653 (88.4)	<0.001	6,331 (87.3)	3,037 (88.3)	0.124
Yes	**2,478 (12.4)**	283 (16.2)	872 (11.6)		922 (12.7)	401 (11.7)	

SD, standard deviation.

^a^Column proportions are shown for the total distribution.

^b^*t*-test was used for age and chi-squared tests were used for other categorical variables.

^c^Infant abnormalities at birth include preterm delivery (<37 gestational week), low birth weight (<2,500 g) and anomalies.

**Table 2.** Factors associated with mothers’ pregnancy intention by parity

Characteristics	Primipara		Multipara	
	
	aOR^a^	(95% CI)	aOR^a^	(95% CI)
**Survey year**				
2012	Reference		Reference	
2013	0.83	(0.73–0.95)^*^	1.28	(1.15–1.42)^*^
2014	1.09	(0.95–1.26)	1.68	(1.51–1.87)^*^
**Maternal characteristics**				
Age at the time of pregnancy, years	0.92	(0.91–0.93)^*^	0.87	(0.86–0.88)^*^
Medical history				
No	Reference		—	—
Yes	0.94	(0.83–1.06)	—	—
Subjective health				
Good	Reference		Reference	
Poor	0.75	(0.66–0.85)^*^	0.81	(0.72–0.92)^*^
Depression symptoms				
No	Reference		Reference	
Yes	0.71	(0.62–0.80)^*^	0.79	(0.71–0.88)^*^
**Residential characteristics**				
Residential region				
Central	Reference		—	—
Coastal	0.89	(0.78–1.02)	—	—
Mountainous	1.16	(0.97–1.39)	—	—
Current evacuation				
No	Reference		—	—
Yes	0.91	(0.72–1.17)	—	—
Living with husband				
Yes	Reference		Reference	
No	0.24	(0.21–0.29)^*^	0.79	(0.64–0.98)
**Obstetrical and parenting characteristics**				
Infertility treatment				
No	—	—	Reference	
Yes	—	—	2.05	(1.66–2.52)^*^
Satisfaction with obstetrical care				
Yes	Reference		Reference	
No	0.89	(0.82–0.95)^*^	0.95	(0.89–1.01)
Maternal confidence				
Yes	Reference		—	—
No	0.88	(0.78–1.00)	—	—
**Infant characteristics**				
Abnormalities at birth^b^				
No	Reference		—	—
Yes	0.72	(0.61–0.84)^*^	—	—

aOR, adjusted odds ratio; CI, confidence interval.

^a^Factors that were significant in univariable analyses were entered into multivariable analyses using binominal logistic regression.

^b^Infant abnormalities at birth include preterm delivery (<37 gestational week), low birth weight (<2,500 g) and anomalies.

^*^P value <0.01.

When limiting data to the first 2 years to include the disaster-related item on concern about radiation contamination of breast milk, the overall proportion of mothers with pregnancy intention was 42.0% and 53.4% among those with (*n* = 188) and without (*n* = 13,302) radiation concern, respectively. Parity-specific multivariable analyses showed that the item on concern about radiation was significantly associated with pregnancy intention only among primiparas (aOR 0.61) (Table 3). Of note, sub-analyses were conducted after eliminating the duplicate live births in each year by keeping the first births in the data to confirm that the statistical significance of variables analyzed in Table 2 and Table 3 did not change, with the exception of survey year among primiparas in Table 2 (aOR 0.89 for 2013, *P* = 0.08).

**Table 3.** Mothers’ pregnancy intention and concern about radiation contamination of breast milk among primiparas in 2012 and 2013

Primipara^b^	Pregnancy intention		aOR^a^	(95% CI)	*P* value

	No	Yes			
Concern about radiation contamination of breast milk					
No (*n* = 6,251)	1,224 (19.6)	5,027 (80.4)	Reference		
Yes (*n* = 69)	25 (36.2)	44 (63.8)	0.61	(0.46–0.80)	<0.001

Multipara^c^					

No (*n* = 7,051)	4,975 (70.6)	2,076 (29.4)	Reference		
Yes (*n* = 119)	25 (70.6)	44 (29.4)	1.02	(0.82–1.26)	0.88

aOR, adjusted odds ratio; CI, confidence interval.

^a^Factors listed in the analysis among primiparas in Table 3 were entered into binominal logistic regression.

^b^Among adjusted variables for primiparas, *P* value was less than 0.01 for survey year (aOR 0.82 for 2013), maternal age (aOR 0.91 for 1-year increase), subjective health (aOR 0.70 for poor), depressive symptoms (aOR 0.63 for positive symptoms), living with husband (aOR 0.24 for not living together), and child abnormality at birth (aOR 0.69 for having abnormality).

^c^Among adjusted variables for multiparas, *P* value was less than 0.01 for survey year (aOR 1.3 for 2013), maternal age (aOR 0.88 for 1-year increase), subjective health (aOR 0.78 for poor), depressive symptoms (aOR 0.72 for positive symptoms), and infertility treatment (aOR 2.07 for receiving the treatment).

The two reasons for not planning a future pregnancy among primiparas were simply “do not want a future pregnancy,” followed by “concerns about age and health condition” across the 3 years (Table 4). Among multiparas, the top reason of “do not want a future pregnancy” increased during the 3 years (*P* for trend <0.001), while the second reason of “busy with care of older sibling(s)” decreased in 2014. As for disaster-related reasons, those who selected “concerns about the impact of radiation contamination” decreased among both primiparas and multiparas, and the proportion of “evacuation or living separately from family” decreased among multiparas in 2013.

**Table 4.** Reasons for no intention of future pregnancy by parity

	*N* (%)^a^			*P* for trend^b^

	2012	2013	2014	
**Primipara**	***(n = 604)***	***(n = 661)***	***(n = 523)***	
Do not want a future pregnancy	214 (35.4)	248 (37.5)	181 (34.6)	0.799
Concerns about age and health condition	195 (32.3)	221 (33.4)	148 (28.3)	0.105
Unstable income	193 (32.0)	189 (28.6)	99 (18.9)	<0.001
Busy with care of the first child	179 (29.6)	219 (33.1)	105 (20.1)	<0.001
Insufficient home support or services	117 (19.4)	127 (19.2)	108 (20.7)	0.583
Concerns about the impact of radiation contamination	91 (15.1)	35 (5.3)	40 (7.7)	<0.001
Evacuation or living separately from family	40 (6.6)	31 (4.7)	26 (5.0)	0.183
Other	27 (4.5)	27 (4.1)	125 (23.9)	<0.001
**Multipara**	***(n = 2,547)***	***(n = 2,551)***	***(n = 2,301)***	
Do not want a future pregnancy	1,431 (56.2)	1,483 (58.1)	1,592 (69.2)	<0.001
Concerns about age and health condition	796 (31.3)	922 (36.1)	717 (31.2)	0.953
Unstable income	615 (24.2)	568 (22.3)	403 (17.5)	<0.001
Busy with care of older sibling(s)	945 (37.1)	954 (37.4)	704 (30.6)	<0.001
Insufficient home support or services	336 (13.2)	329 (12.9)	287 (12.5)	0.415
Concerns about the impact of radiation contamination	370 (14.5)	143 (5.6)	69 (3.0)	<0.001
Evacuation or living separately from family	89 (3.5)	47 (1.8)	33 (1.4)	<0.001
Other	50 (2.0)	53 (2.1)	75 (3.3)	0.001

^a^Reasons were asked only among those with no intention of future pregnancy. Denominators are the total number of respondents as indicated in the top row.

^b^Nonparametric test for trend was used.

The top two service demands across the 3 years among those with pregnancy intention were “child care and pediatric service information” and “nursery school, extended hours, and sick child care” for both primiparas and multiparas (Table 5). As a disaster-related item, mothers who selected “information on radiation health effects” decreased during the 3 years in both groups (*P* for trend <0.001).

**Table 5.** Service demands among mothers with intention of future pregnancy by parity

	*N* (%)^a^			*P* for trend^b^

	2012	2013	2014	
**Primipara**	***(n = 2,657)***	***(n = 2,468)***	***(n = 2,539)***	
Child care and pediatric service information	1,902 (71.6)	1,628 (66.0)	1,804 (71.1)	0.650
Nursery school, extended hours and sick child care	1,720 (64.7)	1,696 (68.7)	1,833 (72.2)	<0.001
Information on radiation health effects	1,558 (58.6)	991 (40.2)	962 (37.9)	<0.001
Maternity and child care leave	1,364 (51.3)	1,365 (55.3)	1,427 (56.2)	0.001
Other	163 (6.1)	144 (5.8)	258 (10.2)	<0.001
**Multipara**	***(n = 943)***	***(n = 1,191)***	***(n = 1,362)***	
Child care and pediatric service information	*600 (63.6)*	*714 (60.0)*	*814 (59.8)*	0.086
Nursery school, extended hours and sick child care	624 (66.2)	787 (66.1)	949 (69.7)	0.069
Information on radiation health effects	572 (60.7)	457 (38.4)	469 (34.4)	<0.001
Maternity and child care leave	453 (48.0)	640 (53.7)	734 (53.9)	0.012
Other	74 (7.9)	97 (8.1)	131 (9.6)	0.131

^a^Service demands were asked only among those with intention of future pregnancy. Denominators are the total number of respondents indicated in the top row.

^b^Nonparametric test for trend was used.

## DISCUSSION

**Figure 3.**
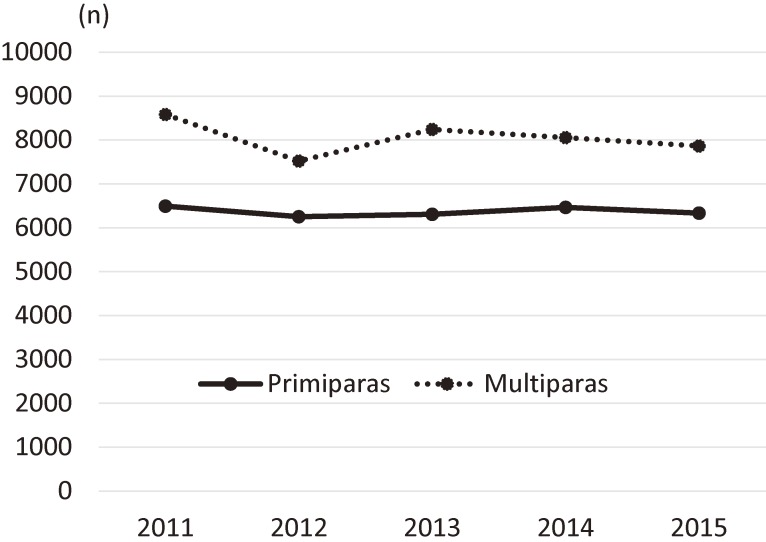
Trends in number of births in Fukushima according to government vital statistics^2^

Using a prefecture-wide survey, we found that the overall intention of future pregnancy among mothers living in Fukushima increased 3 years after the disaster took place in 2011. During these 3 years, the intention of future pregnancy among primiparas continued to decline in 2013, while the intention among multiparas increased immediately after 2012. According to the National Fertility Survey in 2010,^10^ the proportion of couples (married within 5 years) with future pregnancy intention was 89% for those with one child and 48% for those with more than one child, which was higher than what we found in our 2012 data in Fukushima after the disaster (primiparas, 81% and multiparas, 27%). As for subsequent fertility trends, the government’s vital statistics in Fukushima showed an immediate increase in the number of births in 2013 following a steep decline in 2012 among multiparas (Figure 3),^2^ which was consistent with our findings on trends of future pregnancy intention. Taken together, these findings suggest that the overall increase in pregnancy intention in 2014 was due mainly to the increase among multiparas.

In the midst of the confusion around information on the health effects of the Fukushima nuclear accident,^15^ concern about radiation contamination of breast milk was significantly associated with lower intention of future pregnancy among primiparas in 2013 and 2014. Breast milk could be a source of radiation exposure among nursing infants possibly causing thyroid cancer after a nuclear accident. A survey testing breast milk samples from volunteers living within 250 km of the nuclear power plant reported a detectable level of radioiodine in seven of 23 samples in April 2011.^16^ Our data may indicate that women were making fertility decisions in response to the nuclear accident. It may be a rational decision for young mothers to wait for a couple of years before planning their next pregnancy to make sure that their babies were not affected by the disaster. On the other hand, current evacuation as another disaster-related item was not associated with pregnancy intention, which also was reasonable since evacuees were living away from contaminated areas. When examining trends in the reasons behind not planning a future pregnancy, we observed that the proportion of mothers who selected “concerns about the impact of radiation contamination” and “evacuation or living separately from family” decreased from 2013. Similarly, among mothers with pregnancy intention, the proportion of those who selected “information on radiation health effects” as a demanded service decreased. The observed decline in concerns toward radiation exposure may explain the overall increase in future pregnancy intention during the 3 years studied.

Immediately after the Chernobyl nuclear accident in 1986, analyses of demographic data from countries surrounding the most affected areas, namely Italy and Greece, found a temporal decline in live births and a contrasting increase in induced abortions.^17^^,^^18^ On the other hand, similar analyses from Sweden and Finland were inconclusive about whether such demographic trends were induced by the nuclear accident.^19^^,^^20^ Havenaar and van den Brink reviewed the literature on the effects of the stressful experience of toxicological disasters in general, and summarized their profound effects on subjective health and health-related behaviors, especially on reproductive behavior.^21^ Our study is in line with Havenaar and van den Brink’s report showing a possible effect of radiation risk perception on Fukushima mothers’ intention of future pregnancy.

More generally, mothers’ common reasons for not planning a future pregnancy were simply “do not want a future pregnancy” followed by “concerns about age and health condition” for primiparas and “busy with care for older sibling(s)” for multiparas. Among both primiparous and multiparous mothers planning a future pregnancy, they demanded “child care and pediatric service information” and “nursery school, extended hours, and sick child care.” According to the National Fertility Survey in 2010, common reasons for married couples not having their desired number of children were childrearing cost and age, followed by health concerns and childrearing burden.^10^ These data indicate that strategies to leverage the birth rate are improving child care supports and access to these services, especially for couples planning the second or more children, and promoting child bearing at an earlier age. A recent study among middle-aged Japanese found that women with past fertility knowledge gave birth to their first child at an earlier age compared to those without fertility knowledge.^22^ Promoting reproductive education in schools and at home may be needed to increase public fertility knowledge and in turn to promote earlier reproduction.^23^

Further analyses of factors associated with pregnancy intention hint that more practical health service strategies are necessary. Among both primiparas and multiparas, older maternal age, poor subjective health, and having depressive symptoms lowered pregnancy intention. In addition, dissatisfaction with obstetrical care and child abnormalities lowered the pregnancy intention of primiparas, and past infertility treatment increased the intention among multiparas. Age was mentioned by mothers themselves as a factor prohibiting pregnancy intention. Interestingly, all factors other than maternal age suggest that improving perinatal care maybe the key to increasing mothers’ motivation for future pregnancy, which is most directly suggested by the significant association between mothers’ satisfaction with the obstetrical care they received and their future pregnancy intention. Other factors indicate the need to improve services and care for mothers with depression and children with abnormalities, and to support pregnancy intention in those receiving infertility treatment.

Our study has several limitations. First, the response rate was lower than 50%, which limits the generalizability of the obtained findings, especially regarding the proportion of mothers with the intention of future pregnancy. According to government statistics,^1^ the proportion of first births was 44.4% in Fukushima Prefecture during the 3 study years, which was slightly lower than the number (46.5%) from our analysis of FHMS data. Second is that a self-report of future pregnancy intention may not be indicative of actual future pregnancy. We cannot be sure whether a positive intention actually resulted in subsequent childbirth, but 2012 data indicated that those with pregnancy intention were more likely to give birth during the two subsequent years: the proportion of duplicates was 4% among those without pregnancy intention and 20% among those with pregnancy intention. Third is a lack of data on socioeconomic status in the FHMS’s Pregnancy and Birth Survey, although around 20% of mothers marked unstable income as one factor for no intention of future pregnancy. A recent study however reported that a two-child norm, not socioeconomic factors, was associated with probability of childbirth.^24^

In conclusion, this is the first population-based study showing a possible factors underpinning the trends of future pregnancy intention among local mothers at the individual level after the Fukushima nuclear accident. The association was especially strong among primiparas as reflected in their lower pregnancy intention and its association with concern about radiation contamination of breast milk. Whilst concerns toward radiation exposure declined over time, the obtained results further suggest that an increasing pregnancy intention could be maintained by improving obstetrical and mental health care and parenting support. As promoted by a national maternal and child health campaign (“Healthy Parents and Children 21”), “seamless supports” from the antenatal to postnatal phase and partnering of related services and organizations are needed to nurture the next generation.^25^
